# Supplementary material for: ColocML: machine learning quantifies co-localization between mass spectrometry images
Source: Bioinformatics. 2020 Feb 12;36(10):3215–24. doi: 10.1093/bioinformatics/btaa085 (PMC7214035; doi:10.1093/bioinformatics/btaa085)
Supplement: btaa085_Supplementary_Data [file btaa085_supplementary_data.zip › btaa085-Suppl_Data/Supplementary Information (rev2).pdf]

Supplementary Information (Figure S1, Tables S1-S2) for the manuscript

Ovchinnikova et al, ColocML: Machine learning quantifies co-localization between mass spectrometry images

Example results for a target-comparison set

Here, we show an example target-comparison set with the ranking results provided by the rankers as well as by the methods developed in this manuscript. The set is from the dataset 2016-09-21_16h07m37s, submitted to METASPACE by Lennart Huizing, M4I, Maastricht University, from a *Cavia porcellus* intestinal tissue section analyzed by MALDI-FTICR in the positive mode with the DHB matrix (METASPACE URL). Supplementary Figure S1 shows the target ion image. Supplementary Tables S1 and S2 show the comparison ion images, the values of the measures requiring no learning and the values of the deep learning models.

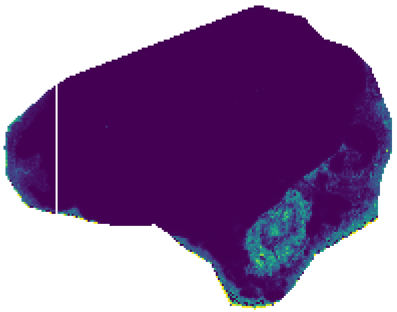

Supplementary Figure S1. The target image for the ion $C_{16}H_{18}O_9S$+Na from an example target-comparison set.

Comparison ion images	GS rank	Cosine	Tfidf-cosine	Pearson	Spearman	SSIM
C21H14O10+Na 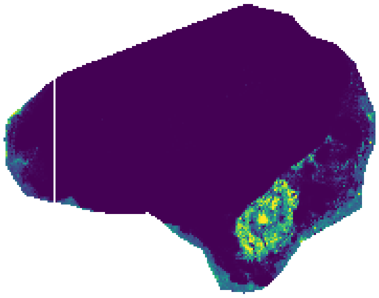	0.0	0.939	0.857	0.934	0.794	0.399
C39H79N2O6P+H 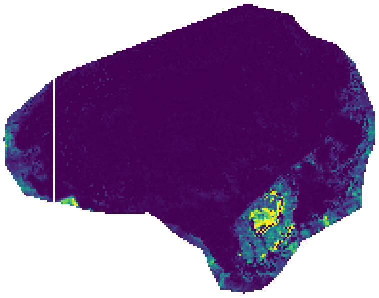	0.666	0.778	0.656	0.759	0.698	0.276
C39H79N2O6P+K 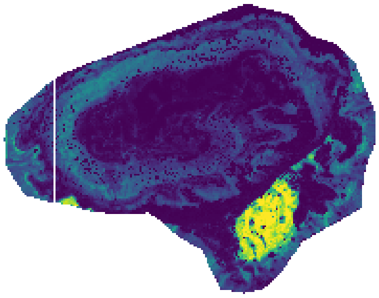	4.0	0.713	0.731	0.687	0.465	0.307
C47H91N2O6P+K 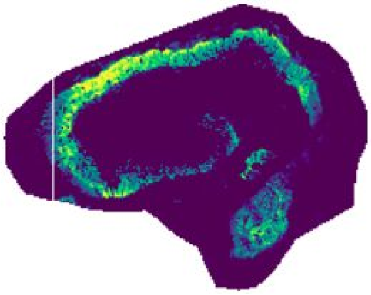	4.333	0.769	0.471	0.747	0.810	0.280
C37H68O4+H	4.333	0.238	0.286	0.152	0.169	0.225

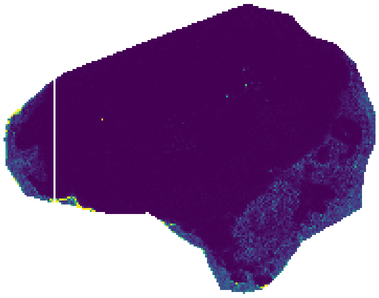						
C26H54NO7P+Na 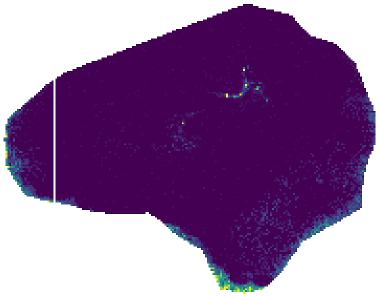	6.333	0.571	0.144	0.553	0.381	0.276
C42H82NO8P+H 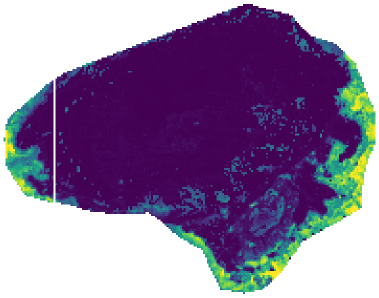	6.666	0.591	0.296	0.546	0.705	0.287
C40H80NO8P+H 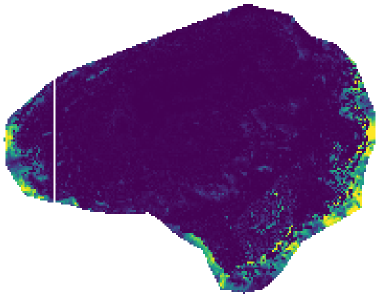	7.0	0.566	0.204	0.529	0.551	0.260
C42H80NO8P+K	8.333	0.527	0.386	0.464	0.490	0.273

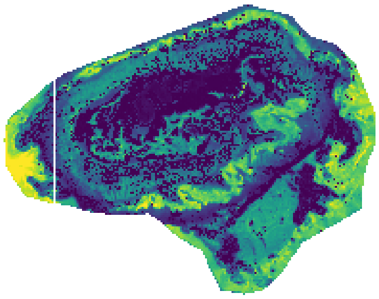						
C44H82NO8P+Na 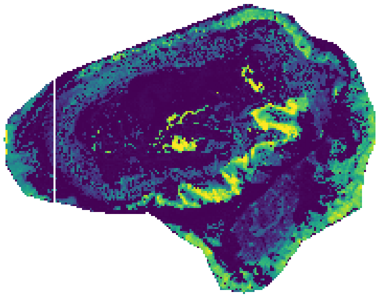	9.0	0.418	0.205	0.333	0.390	0.310

Supplementary Table S1. Average experts rank from the gold standard (GS) (ranging from 0 to 10; low rank corresponding to higher perceived co-localization) and co-localization measures requiring no learning (ranging from 1 to 0; higher value corresponding to higher estimated co-localization) for the comparison images from the selected target-comparison set from the gold standard set.

Comparison ion images	GS rank	Pi model	Xception model	Unsupervised UMAP	UMAP+ GBT	Mu model
C21H14O10+Na 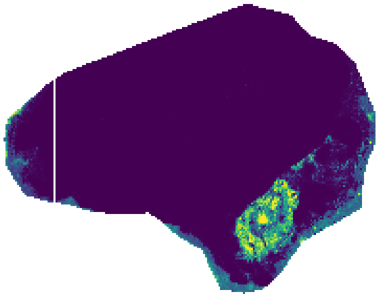	0.0	2.404	1.787	1.350	2.444	3.353
C39H79N2O6P+H 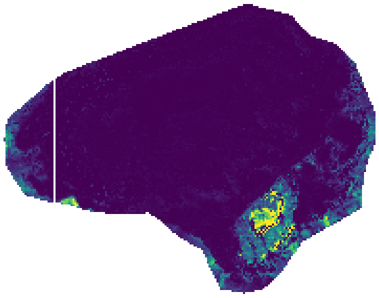	0.666	2.049	1.291	0.225	2.396	5.315
C39H79N2O6P+K 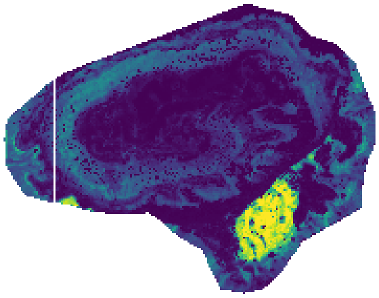	4	7.203	7.592	5.525	4.460	7.144
C47H91N2O6P+K 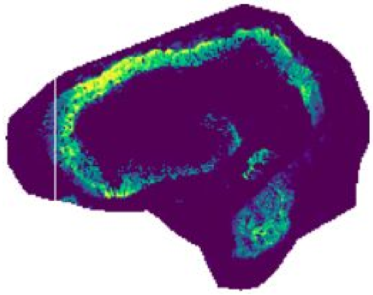	4.333	7.468	8.210	9.000	7.973	8.590
C37H68O4+H	4.333	3.704	3.499	2.775	3.698	3.834

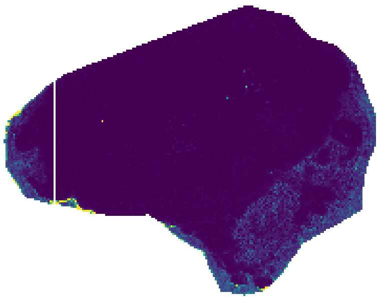						
C26H54NO7P+Na 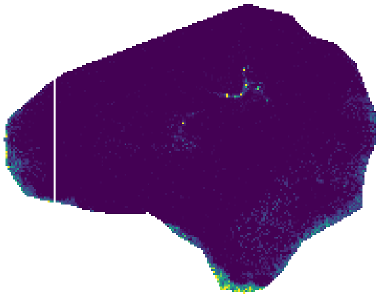	6.333	5.860	5.553	5.300	6.032	3.458
C42H82NO8P+H 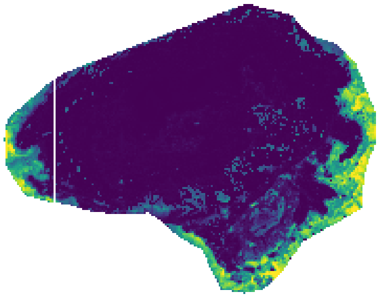	6.666	5.689	6.291	3.650	3.167	4.481
C40H80NO8P+H 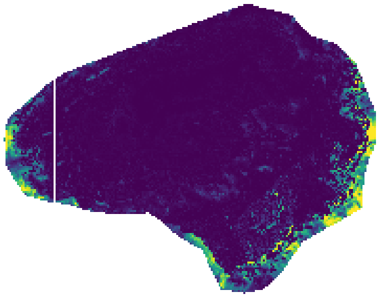	7.0	4.450	4.085	2.550	3.749	4.969
C42H80NO8P+K	8.333	8.162	8.037	6.625	6.103	7.573

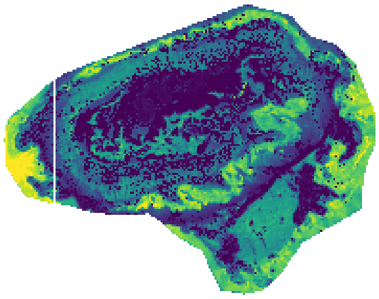						
C44H82NO8P+Na 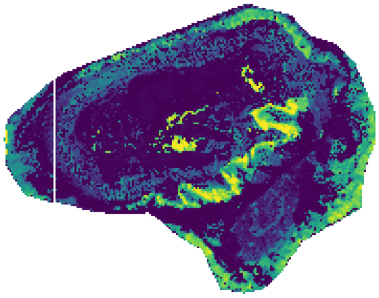	9.0	8.310	9.575	8.000	6.130	7.869

Supplementary Table S2. Average experts rank from the gold standard (GS) (ranging from 0 to 10; low rank corresponding to higher perceived co-localization) and co-localization measures based on deep learning (ranging from 0 to 10; low value corresponding to higher estimated co-localization) for the comparison images from the selected target-comparison set from the gold standard set.
